# Advances in the Application of Radionuclide-Labeled HER2 Affibody for the Diagnosis and Treatment of Ovarian Cancer

**DOI:** 10.3389/fonc.2022.917439

**Published:** 2022-06-15

**Authors:** Xianwen Hu, Dandan Li, Yujie Fu, Jiashen Zheng, Zelong Feng, Jiong Cai, Pan Wang

**Affiliations:** ^1^ Department of Nuclear Medicine, Affiliated Hospital of Zunyi Medical University, Zunyi, China; ^2^ Department of Obstetrics, Zunyi Hospital of Traditional Chinese Medicine, Zunyi, China; ^3^ Research and Development Department, Jiangsu Yuanben Biotechnology Co., Ltd., Zunyi, China

**Keywords:** human epidermal growth factor receptor, ovarian cancer, radionuclide, HER2 affibody, molecular probes

## Abstract

Human epidermal growth factor receptor 2 (HER2) is a highly expressed tumor marker in epithelial ovarian cancer, and its overexpression is considered to be a potential factor of poor prognosis. Therefore, monitoring the expression of HER2 receptor in tumor tissue provides favorable conditions for accurate localization, diagnosis, targeted therapy, and prognosis evaluation of cancer foci. Affibody has the advantages of high affinity, small molecular weight, and stable biochemical properties. The molecular probes of radionuclide-labeled HER2 affibody have recently shown broad application prospects in the diagnosis and treatment of ovarian cancer; the aim is to introduce radionuclides into the cancer foci, display systemic lesions, and kill tumor cells through the radioactivity of the radionuclides. This process seamlessly integrates the diagnosis and treatment of ovarian cancer. Current research and development of new molecular probes of radionuclide-labeled HER2 affibody should focus on overcoming the deficiencies of non-specific uptake in the kidney, bone marrow, liver, and gastrointestinal tract, and on reducing the background of the image to improve image quality. By modifying the amino acid sequence; changing the hydrophilicity, surface charge, and lipid solubility of the affibody molecule; and using different radionuclides, chelating agents, and labeling conditions to optimize the labeling method of molecular probes, the specific uptake of molecular probes at tumor sites will be improved, while reducing radioactive retention in non-target organs and obtaining the best target/non-target value. These measures will enable the clinical use of radionuclide-labeled HER2 affibody molecular probes as soon as possible, providing a new clinical path for tumor-specific diagnosis, targeted therapy, and efficacy evaluation. The purpose of this review is to describe the application of radionuclide-labeled HER2 affibody in the imaging and treatment of ovarian cancer, including its potential clinical value and dilemmas.

## Introduction

Ovarian cancer is the disease with the highest mortality rate after cervical cancer secondary to female reproductive tract malignant tumors; in 2020, 313,959 new patients with ovarian cancer and 207,252 new deaths from ovarian cancer were reported worldwide. Although ovarian cancer is generally more common in developed countries, studies have shown an increasing incidence in China (1, 2). Several risk factors for ovarian cancer include genetics, reproduction, sex hormones, and lifestyle behaviors (3). Specifically, a family history of ovarian cancer in first- or second-degree relatives, polycystic ovary syndrome, endometriosis, gynecological inflammation, increased estrogen/androgen, high-fat diet, etc. are associated with a high risk of cancer. However, fertility, contraception including the intrauterine device and oral contraceptives, tubal ligation, and breastfeeding can reduce the risk of ovarian cancer (4–14). Ovarian cancer has an insidious onset and lacks specific clinical symptoms in the early stage, and the clinical diagnostic process is relatively unsatisfactory. Approximately 60% of patients with ovarian cancer are in the advanced stage of the disease by the time symptoms are recognized or medical help is sought. Therefore, the prognosis of patients is poor, with a 5-year survival rate of less than 30% (15, 16).

## Overview of HER2 and Affibody

Human epidermal growth factor receptor 2 (HER2), also known as receptor tyrosine protein kinase erbB-2, is a member of the epidermal growth factor receptor family (17). The oncogenic mechanisms of HER2 include inhibiting tumor cell apoptosis, increasing tumor cell invasiveness, promoting tumor cell proliferation, and promoting tumor angiogenesis and lymphangiogenesis (18). HER2 is rarely expressed in normal ovarian epithelial cells, but is highly positive in epithelial ovarian cancers, including 45.5% of mucinous carcinomas, 41.7% of clear cell carcinomas, and 17.5% of serous carcinomas (19). Studies confirmed that the positive expression of HER2 is significantly correlated with patient prognosis (20, 21). Therefore, it is significantly important to obtain HER2-positive expression accurately and effectively for the precise diagnosis and targeted therapy of HER2-positive ovarian cancer.

The use of multiple methods to detect the presence of relevant mutations in tumor specimens and the optimal targets of targeted therapy, which are useful for prognosis and treatment, is the best strategy to strengthen and improve patients with ovarian cancer (22–24). Clinically, the state of HER2 expression is often determined by immunohistochemistry (IHC) and nucleic acid fluorescence *in situ* hybridization (FISH) on biopsy tissue (25). IHC uses the principle of specific binding of antigen and antibody, and determines the antigen in tissue cells through a chemical reaction to develop the color of the chromogenic reagent labeled with the antibody. However, the stability of this method is poor, and various technical variables, different antibody sensitivities, and resulting evaluation systems during the operation may affect the test results (26). FISH is a molecular cytogenetic technique that uses fluorescent probes that bind only to parts of chromosomes with a high degree of sequence complementarity. DNA is analyzed under the microscope by fluorescence detection (25, 26). By using gene-specific DNA probes to determine the copy number of the HER2 gene, the detection results are reliable due to the relative stability of the DNA; however, this method is expensive and technically complicated. Furthermore, the expression of the protein is regulated by many factors; therefore, the HER2 gene is regulated by many factors and the amplification of the HER2 gene is not always consistent with the overexpression of protein (27, 28). In addition, both IHC and FISH are invasive tests, which only reflect the local situation of the biopsy tissue. These tests cannot reflect the HER2 expression of the tumor as a whole and other metastases. Hillig et al. showed that the misdiagnosis rate of HER2 expression measured by IHC or FISH is 20% (29). Single photon emission computed tomography (SPECT) and positron emission tomography (PET) imaging can obtain functional information about the organization of biological chemistry. Advantages of these methods include noninvasiveness, accuracy, and safety. become one of the hot spot of the HER2 receptor positive tumors. The positive rate of HER2 expression in ovarian cancer is low. Although the nuclear medicine molecular probe targeting the HER2 receptor is not suitable for the screening and diagnosis of ovarian cancer directly, it can be used as an important complementary means to IHC or FISH. This method can obtain a general expression of HER2 positive, including primary tumor location, the extent of the tumor invasion into the surrounding tissue, and the detection of metastasis sites. In addition, targeting the HER2 receptor helps clinicians monitor the efficacy of HER2-targeted therapy in patients and facilitates stratified patient studies, laying a solid foundation for the integration of ovarian cancer diagnosis and treatment.

**Figure 1:**
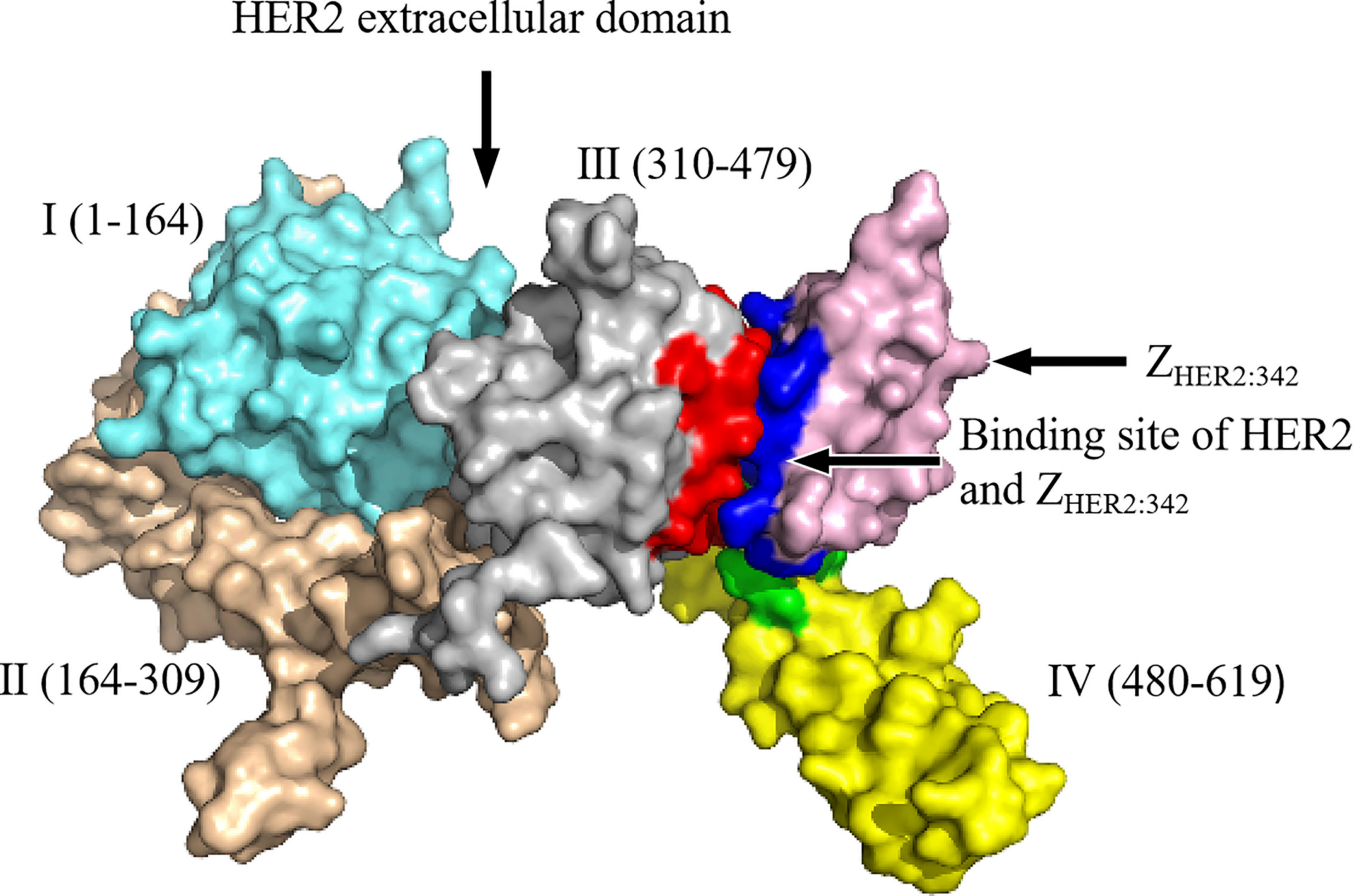
The schematic representation of HER2 affibody and the molecular interactions with its targets: the HER2 extracellular region is further divided into domain I (1–164, sky blue), domain II (164–309, brown), domain III (310–479, silver gray), and domain IV (480–619, yellow). Pink represents Z_HER2:342_; dark blue represents Z_HER2:342_ binding residues; red represents residues on domain III that interact with Z_HER2:342_; green represents residues on domain IV that interact with Z_HER2:342_. The detailed amino acid sequences of HER2 extracellular domains I–IV are presented in **Supplementary Table 1**.

Affibody is an artificial protein molecule with a single-chain structure, and is a new type of protein ligand that evolved from the B segment of the immunoglobulin binding region of *Staphylococcus* protein A, with a relative molecular weight of 6.5 kDa. Affibody has the ability to bind to other proteins (30). SPA is a cell wall protein of type A *Staphylococcus aureus*, containing 7 domains (S, E, D, A, B, C, and X), among which the B domain contains 58 amino acid residues and is the main functional fragment that mediates the binding of SPA to the FC segment of IgG (except IgG3) (31). These amino acid defects constitute three α-helix structures. In 1995, to improve the chemical stability of the affinity body, the Swedish scholar Nord et al. replaced the 29th glycine of the B domain with alanine and renamed it as the Z domain (32). The amino acids at 13 positions in the first and second helices of the Z domain are Q9 (glutamine), Q10 (glutamine), N11 (asparagine), F13 (benzene), Y14 (tyrosine), L17 (leucine), and H18 (histidine) of the first α-helix, and (alanine), E24 (glutamic acid), E25 (glutamic acid), R27 (arginine), N28 (asparagine), Q32 (glutamine), and K35 (lysine) of the second α-helix, which have no obvious effect on the higher-order structure of the Z domain. The Z domain retains the binding function of the Fc segment (33). Random replacement of these 13 amino acid codons with degenerate codon NNK (K = G, Guanylate or T, Thymine, including 32 codons and 20 amino acids) can theoretically generate 3,213 gene sequences and 2,013 amino acid sequences; these constitute the affibody library. The affibody library can basically be combined with any protein molecule, and the affinity body of a certain protein molecule can be obtained after screening (32, 34). In addition to HER2, a variety of proteins such as fibrinogen, transferrin, tumor necrosis factor Q, interleukin-8, CD28, human serum albumin, IgA, IgE, IgM, and epidermal growth factor receptor (EGFR) have been discovered, and their affinity is in the range of μmol/L to pmol/L according to the characteristics of the bound proteins and the structure of the affibody (35). Due to the small size and high affinity of HER2 affibody that can specifically bind to HER2 receptor both *in vitro* and *in vivo*, rapid clearance from blood and non-targeted tissues, ease of structural modification, and selectivity for cancer-related targets, this affibody is suitable as a radionuclide molecular probe and has excellent potential for use in the diagnosis and treatment of cancer (36–41). The schematic representation of the HER2 affibody and its interaction with the target molecule, HER2, is shown in **Figure 1**.

**Figure 2:**
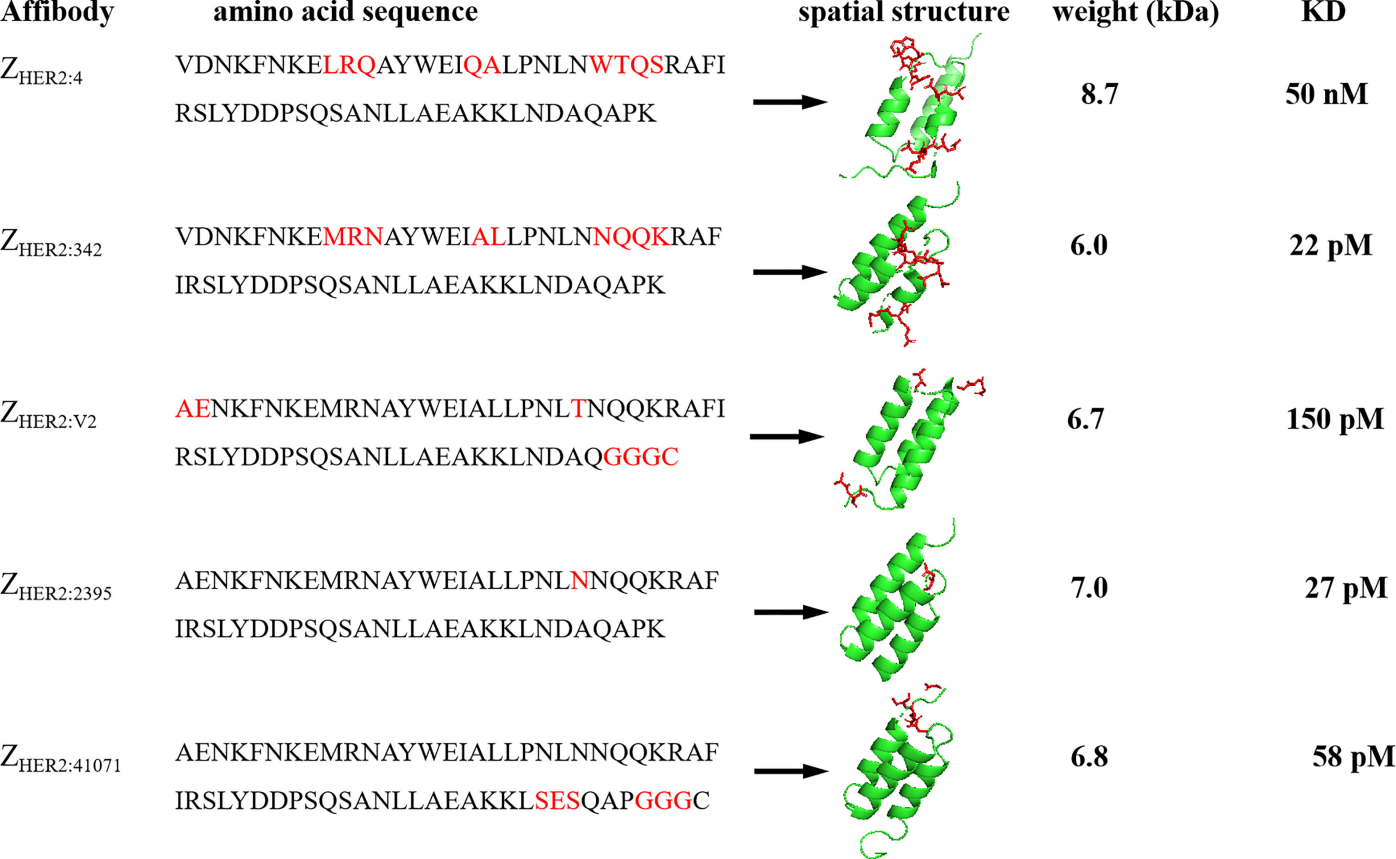
The evolution process of affibody. Red indicates the modified amino acid sequence.

The first-generation HER2 affibody Z_HER2:4_ has a low affinity; therefore, researchers combined its head and tail to form a dimer (Z_HER2:4_)2, and its dissociation equilibrium constant KD value changed from the original 50 nM to 3 nM, leading to obvious improvement in its affinity (42, 43). The second-generation HER2 affibody is mainly Z_HER2:342_, which has a triple-helix structure through modification of the first generation; its affinity is 2,200 times that of the first generation (44). The third-generation affibody is mainly Z_HER2:2395_, which is made by introducing a cysteine at the carboxy terminus of Z_HER2:342_ to obtain a unique attachment site, using bifunctional chelators, linking molecules or other thiol-reactive carriers, such as maleimide or iodoacetamide, which can be directionally coupled to this particular cysteine. Z_HER2:2395_ has an affinity of approximately 27 pM, which is comparable to that of the second generation (45, 46). A recent study reported the replacement of the -NDA-sequence near the C segment of the affibody Z_HER2:2395_ with the -SES-sequence, which further improved the stability and hydrophilicity of the affibody, resulting in an affinity that is close to Z_HER2:2395_ (47). The detailed evolution process of the affibody is shown in **Figure 2**.

## Overview of Radionuclide-Labeled HER2 Affibody Molecular Probes

Molecular probes targeting HER2 affibody can evaluate the expression status of tumor HER2 receptors and predict early efficacy after treatment, and significant progress has been made in the research on the integration of tumor diagnosis and treatment. The main applications of radionuclide-labeled HER2 molecular probes are summarized in **Table 1**.

**Table 1:** Overview of the major applications of radionuclide-labeled HER2 affibody molecular probes.

Radioisotope	T1/2	Diagnostic/therapy	Representative molecular probes	Study object	First posted	Phase	References
^111^In	2.81 days	SPECT	^111^In-ABY-002	Ovarian cancer	05/2006	Preclinical	(48–52)
				Breast cancer	07/2007	I	(53)
			^111^In-DOTA-Z_HER2:342_	Ovarian cancer	08/2008	Preclinical	(49)
			^111^In-Z_HER2:2395_	Prostate cancer	05/2012	Preclinical	(54)
^99m^Tc	6.02 h	SPECT	^99m^Tc-Z_HER2:342_	Ovarian cancer	12/2006	Preclinical	(55–59)
			^99m^Tc-Z_HER2:V2_	Lung cancer	11/2018	Preclinical	(60, 61)
				Ovarian cancer	02/2011	Preclinical	(62, 63)
			^99m^Tc-Z_HER2:41071_	Ovarian cancer	03/2021	Preclinical	(47)
			^99m^Tc-ABH2	Breast cancer	06/2018	I	(64)
^125^I	60 days	SPECT/therapy	^125^I-Z_HER2:4_	Ovarian cancer	06/2005	Preclinical	(42, 43)
			^125^I-IPEM-Z_HER2:2395_	Ovarian cancer	01/2015	Preclinical	(65)
^18^F	109.8 min	PET	^18^F-Z_HER2:342_	Breast cancer	06/2012	Preclinical	(66)
				Ovarian cancer	12/2007	Preclinical	(67, 68)
^68^Ga	68 min	PET	^68^Ga-DOTA-MUT-DS	Ovarian cancer	08/2009	Preclinical	(69)
			^68^Ga-ABY-002	Breast cancer	07/2007	I	(53)
			^68^Ga-ABY-025	Breast cancer	05/2013	I/II	(70, 71)
^64^Cu	12.7 h	PET/therapy	^64^Cu-Z_HER2:342_	Ovarian cancer	10/2019	Preclinical	(72)
^188^Re	17 h	Therapy	^188^Re-Z_HER2:V2_	Ovarian cancer	10/2014	Preclinical	(73, 74)
^177^Lu	6.7 days	Therapy	^177^Lu-Z_HER2:342_	Ovarian cancer	03/2007	Preclinical	(75)

### HER2 Affibody Molecular Probes as SPECT Tracers

**[^111^In]:** Indium-11 (^111^In) is a pure γ-ray emitter with a half-life of 2.81 days, making it an ideal radionuclide tracer for SPECT imaging. Benzyl-dtpa,-CHX-A”-DTPA (diethylenetriaminepentaacetic acid) and DOTA (1,4,7,l0-tetraazacyclodo-decane-*N,N,N,N*-tetra acetic acid) have been used as chelating agents for affibody Z_HER2:342._ When labeled with ^111^In, which showed a good labeling yield (>95%) and a high affinity (21-65 pM), the molecular probe with specific uptake by the SKOV3 ovarian cancer xenograft tumor tissues with high HER2 receptor expression showed high tumor targeting binding properties (48–52). Unfortunately, in addition to tumor uptake of these molecular probes, renal uptake is also high, increasing the potential for renal toxicity. Based on this disadvantage, Honarvar et al. (76) designed a 15-mer HP1 PNA recognition tag and a complementary HP2 hybridization probe ^111^In-Z_HER2:342_-SR-HP1 to reduce the uptake of the molecular probe in the kidney. Moreover, a number of studies have used DOTA as a chelating agent to bind to the affibodies Z_HER2:342min_, Z_HER2:2395_, and Z_HER2:2395_-Cys, and then labeled with the radionuclide ^111^In. This method shows good potential for targeted imaging (77–80). However, one disadvantage of using DOTA as a chelator is that the liver uptake is relatively high. Researchers then compared the results of using DOTAGA (2-[1,4,7,10-Tetraazacyclododecane-4,7,10-tris(t-butyl acetate)]-pentanedioic acid-1t-butyl ester) instead of DOTA as a chelator, and found that the change improved the biodistribution of molecular probes *in vivo*, reducing uptake by the liver (81).

**[^99m^Tc]:** Technetium-99m (^99m^Tc) emits γ-rays of 140 keV and has a half-life of 6.02 h. It can be obtained by the ^99^Mo-^99m^Tc generator and is inexpensive; therefore, it is widely used in clinical and basic research. An early study used the indirect labeling method to add chelating agent maGGG (mercaptoacetyl-glycyl-glycyl-glycyl) to the N-terminal of the affibody Z_HER2:342_, and successfully labeled affibody Z_HER2:342_ with ^99m^Tc. The researchers further completed a series of *in vivo* biodistribution and imaging studies, and the results showed that the molecular probe was specifically absorbed by SKOV3 ovarian cancer xenograft tumor with high expression of the HER2 receptor and high targeting ability (79). However, the liver and gastrointestinal tract uptake of this molecular probe is high, thus affecting the detection rate of abdominal lesions. Subsequently, the research team replaced the chelating agent maGGG with maGSG (mercaptoacetyl-glycyl-seryl-glycyl), maG(D-S)G (mercaptoacetyl-glycyl-D-seryl-glycyl), and maSSS (mercaptoacetyl-seryl-seryl-seryl), respectively, and labeled it with ^99m^Tc. Results showed that when the glycine residue in the chelating agent maSSS was replaced with a hydrophilic serine amino group, the hydrophilic activity of the chelating agent maGGG was increased. Therefore, the uptake of the molecular probe in the liver and gastrointestinal tract was significantly reduced, resulting in a significant increase in the detection rate of abdominal tumor lesions. However, the excretion route was shifted from the liver to the kidney, increasing the potential for kidney toxicity (80). To overcome this shortcoming, the team further optimized the study design and compared the ^99m^Tc labeling studies using maGEG (mercaptoacetyl-Gly-Glu-Gly), maEEE (Mercaptoacetyl-Glu-Glu-G1u), maESE (mercaptoacetyl-glutamyl-seryl-glutamyl), maEES (mercaptoacetyl-glutamyl-glutamyl-seryl), maSEE (mercaptoacetyl-seryl-glutamyl-glutamyl), maSKS (mercaptoacetyl-seryl-lysyl-seryl), and maKKK (mercaptoacetyl-trilysyl) as chelators. The results showed that the use of the molecular probes ^99m^Tc-maEEE-Z_HER2:342_, ^99m^Tc-maSKS-Z_HER2:342_, and ^99m^Tc-maESE-Z_HER2:342_ with maEEE, maSKS, and maESE as chelators significantly reduced the radioactive uptake in the kidney. In addition, the radioactive uptake of ^99m^Tc- maESE-Z_HER2:342_ was found to be the most obvious, further improving the abdominal image quality (55, 56, 81). Furthermore, other studies introduced a chelating agent at the C-terminus or/and N-terminus of the affibody Z_HER2:342_ for ^99m^Tc labeling, and obtained molecular probes ^99m^Tc-(HE)_3_-Z_HER2:342_-GGGC, ^99m^Tc-Z_HER2:342_-GGGC, ^99m^Tc-Z_HER2:V2_, etc. The researchers performed *in vivo* biodistribution and imaging studies, and results showed that these molecular probes are stable *in vitro* and *in vivo*, with high specificity and high targeting (82–84).

**[^123^I/^125^I]:** Iodine-123 (^123^I) has a half-life of 13.3 h and can emit 159 keV γ rays, which is suitable for SPECT imaging. ^125^I has a long half-life (59.4 days) and is a convenient replacement nuclide usually used in the development of radioactive iodization technology instead of ^123^I and ^124^I (85). An early study used ^125^I labeling for the affibody (Z_HER2:4_)2, but SPECT imaging showed high radioactivity retention in the kidney and liver in addition to tumor uptake. This resulted in substandard image quality (86). To address the prevention of affibody destruction by radionuclides upon labeling, further studies were performed with iodobenzoate (PIB) and [4-isothiocyanate-amino]-undecahydro-o-dodecaborate (DABI) as chelators to label affibody molecule Z_HER2:342_ with ^125^I, and molecular probes ^125^I-PIB-Z_HER2:342_ and ^125^I-DABI-Z_HER2:342_ were prepared. Results showed that ^125^I-PIB-Z_HER2:342_ expressed high contrast imaging and low renal radioretention of HER2 in the mice bearing SKOV-3 xenografts; however, a disadvantage was that the molecular probe did not bind specifically to the tumor (44, 87). Subsequently, the team used HPEM(4-hydroxyphenyl-ethyl-maleimide) as a chelating agent to design and synthesize the molecular probe ^131^I/^125^I-HPEM-Z_HER2:342_-C. Compared with the previous molecular probe ^125^I-PIB-Z_HER2:342_, HPEM provided a site-specific conjugate for indirect radioiodination of cysteine-containing affibody Z_HER2:342_, and preserved the specificity of binding to HER2-expressing cells (88). Several studies have shown that the labeling strategy of using HPEM as a chelator further reduces renal uptake, but increases hepatobiliary and gastric uptake; thus, this strategy is detrimental to the detection of abdominal metastases (85, 87, 88). To solve this problem, the HEHEHE sequence was recently introduced into the amino terminal of the affibody, and the molecular probe ^125^I-PIB-(HE)_3_-G_3_ was prepared by using PIB as a chelating agent. This process reduces the radioactive retention of the liver and kidney while maintaining the highly specific binding of tumor cells, and enables the obtainment of high contrast imaging of tumors expressing HER2 receptor *in vivo* (89).

### HER2 Affibody Molecular Probes as PET Tracers

PET can provide molecular information such as function and metabolism, and has important clinical value in early diagnosis, staging and restaging of tumors, finding primary tumor or metastases, guiding tumor treatment, evaluating treatment efficacy, and predicting recurrence (90). Compared with SPECT imaging, PET imaging has the advantages of high spatial resolution, good sensitivity, and quantification (91). The radionuclide fluorine-18 (^18^F), gallium-68 (^68^Ga), copper-64 (^64^Cu), rhenium-186/188 (^186/188^Re), and other labeled HER2 affibodies have been used in preclinical research on ovarian cancer, showing good prospects for clinical application (54, 67, 68, 78, 92–97).

**[^18^F]:** The half-life of ^18^F is 109.8 min, and the positron energy produced by ^18^F is low (average of 0.25 MeV). Its annihilation distance in tissues is short (approximately 2.4 mm), which enables the obtainment of high-resolution images. Therefore, ^18^F is considered as the most ideal nuclide for PET imaging. An early study used FBO (N-[4-flfluorobenzylidene]oxime) as a chelating agent to label the affibody Z_HER2:477_ to obtain the molecular probe ^18^F-FBO-Z_HER2:477_. The results of animal PET imaging showed that the molecular probe can specifically bind to the HER2 receptor *in vitro* and *in vivo*, and obvious radionuclide uptake can be seen in the SKOV3 tumor tissue. However, due to the large amount of radioactive retention in the liver and kidney, the molecular probe is not clinically applicable (94). Kramer-Marek et al. used N-[2-(4-fluoro-benzamido)ethyl] maleimide (FBEM) as a chelating agent to prepare the molecular probe ^18^F-FBEM-Z_HER2:342_, and animal PET imaging results showed that the molecular probe had high radiation uptake and high image contrast in mice bearing SKBR3 and SKOV-3 xenografts with high HER2 expression, but no obvious uptake in mice bearing MDA-MB-468 xenografts with low HER2 expression. However, the high radioactive uptake in the kidney and bone also limits its clinical application (67). Recently, researchers have used FET (fluoroethyl-L-tyrosine) as a linker to prepare a molecular probe [^18^F]FET-Z_HER2:342_, and a preclinical study has shown that the molecular probe specifically binds to tumors and has a lower radioactive uptake in the liver. Unfortunately, its radiochemical yield is relatively low (68). Moreover, different methods of ^18^F labeling for Z_HER2:2395_ and Z_HER2:2891_ have been reported, but their clinical transformation has been hindered by a large amount of radioactive retention in the kidney or the complex manufacturing process and low yield of molecular probes (62, 63, 73 ).

**[^68^Ga]:** The half-life of ^68^Ga is 68 min, and the positron decay rate is 89%. ^68^Ga can be prepared by a ^68^Ge-^68^Ga generator. It is suitable for labeling small molecules that can rapidly distribute *in vivo* and reach the target, and high-quality images can be obtained approximately 1 h after intravenous injection. An early animal study of PET imaging used DOTA as a chelating agent to label the HER2 affibody Z_HER2:342_ and the small-molecule protein MUT-DS of HER2, respectively, with ^68^Ga (69). The results showed that the tumor tissue with high expression of HER2 receptor showed obvious radionuclide aggregation, which was quickly cleared in the blood, with low background and high image quality. However, the disadvantage was that there was also a high radioactive uptake in the kidney. Recently, a number of studies have successively used DOTA, NOTA (1,4,7-triazacyclononane-*N,N,N*-triacetic acid), and NODAGA [1-(1,3-carboxypropyl)-4,7-carboxymethyl-1,4,7-triazacyclononane] as chelating agents to label the affibodies Z_HER2:S1_, Z_HER2:2395_, and Z_HER2:2891_, respectively, and performed molecular probe research *in vivo* and *in vitro*. The results showed that NODAGA as a chelator for ^68^Ga-labeled synthesis of molecular probes provided the best tumor-to-organ ratio, and was the best chelator for ^68^Ga-labeled HER2 affibody, indicating excellent prospects for clinical application (81, 92, 93).

**[^64^Cu]:** ^64^Cu can simultaneously release β^-^ with a maximum energy of 580 keV and β^+^ with a maximum energy of 656 keV, which can be used for PET imaging and radionuclide therapy. The half-life of ^64^Cu is 12.7 h, and it can be produced by nuclear reactors and medical accelerators (98, 99). Researchers first used DOTA as a chelating agent to label the affibody Z_HER2:477_ with ^64^Cu and performed PET imaging studies in tumor-bearing nude mice with high HER2 receptor expression. The results showed that the molecular probe can be significantly taken up by tumor tissue. However, the disadvantage is that there is also a large amount of aggregation in the liver and kidney, and the uptake of radioactivity in liver and kidney is significantly higher than that in tumor tissue, thus affecting the detection of abdominal lesions (100). Subsequently, the research team further introduced cysteine into different positions of the affibody Z_HER2:342_ for ^64^Cu labeling to prepare molecular probes ^64^Cu-DOTA-Cys-Z_HER2:342_, ^64^Cu-DOTA-Z_HER2:342_(Cys39), and ^64^Cu-DOTA-Z_HER2:342_-Cys. All three probes showed good affinity and stability in *in vitro* studies, and ^64^Cu-DOTA-Cys-Z_HER2:342_ had the highest affinity and *in vivo* stability. However, the problem of high renal radioactivity retention remained unresolved (72). In addition, some researchers conducted *in vitro* and *in vivo* studies and compared the use of NOTA and NODAGA as chelators for ^64^Cu labeling of affibodies. The results showed that the tumor-to-organ ratio was higher when NODAGA was used as a chelator than when NOTA was used as a chelator. The disadvantage was that the molecular probes had higher radioactive retention in the kidney and bone marrow (101). Therefore, research of ^64^Cu-labeled HER2 affibody molecular probe needs to be further optimized and perfected.

**[^186/188^Re]:** The two isotopes of rhenium, ^186^Re and ^188^Re, can simultaneously emit γ rays and β rays, enabling their use for both imaging and therapy. ^186^Re is produced by the reactor and has a half-life of 3.72 days. ^186^Re can emit β rays with a maximum energy of 1.08 MeV and γ rays with an energy of 155 keV at a time. ^188^Re has a half-life of 17.0 h, can be produced by a ^188^W/^188^Re generator, and can emit β rays with a maximum energy of 2.1 MeV and γ rays with an energy of 137 keV. Orlova et al. (102) first used maGGG and maGSG as chelators to label affibody Z_HER2:342_ with ^186^Re and compared the resulting molecular probe with the ^99m^Tc-labeled molecular probe. The results showed that the molecular probe ^186^Re-maGSG-Z_HER2:342_ showed obvious radioactive uptake in the tumors of mice bearing SKOV3 xenografts at 4 h after injection. Compared with the ^99m^Tc-labeled molecular probe, the ^186^Re-labeled molecular imaging probe significantly reduces the renal uptake; its renal uptake is only 1/4 of the ^99m^Tc-labeled molecular probe. However, the excretion of molecular probes through the hepatobiliary tract leads to relatively high uptake in the liver, which may affect the detection of liver lesions. Subsequently, the same research team attached different amino acid sequences to the carboxy-terminus to label the affibodies with ^188^Re and compared them *in vitro* and *in vivo*. The results showed that the uptake of the molecular probe ^188^Re-Z_HER2:V2_ with GGGC sequence linked to the carboxyl terminus was significantly higher than that of any organ tissue (including liver and kidney) at 4 h after injection. Furthermore, the uptake of the kidney was 5 times lower than that of the tumor, indicating that this molecular probe is a promising candidate for imaging and treatment of tumors with high expression of HER2 receptor (73, 74).

In addition, preclinical studies of PET molecular probes labeled to HER2 affibody by radionuclides such as astatine-211 (^211^At), scandium-44 (^44^Sc), zirconium-89 (^89^Zr), and carbon-11 (^11^C) through different chelating agents in SKOV3 ovarian tumors with high HER2 expression have also been reported in the literature, and the results show good potential for clinical application (103–106).

### Radionuclide-Labeled HER2 Affibody as Targeted Therapeutic Probes

Ovarian cancer is traditionally treated with radical tumor resection and platinum-paclitaxel chemotherapy. Most tumors eventually become resistant to cytotoxic chemotherapy, leading to disease recurrence and progression (107). Recent studies have shown significant efficacy of poly (ADP-ribose) polymerase (PARP) inhibitors in patients with epithelial ovarian cancer, especially in patients with germline breast-related cancer antigens 1 and 2 (BRCA1/2) mutations. However, its clinical application value in patients without BRCA1/2 mutation remains controversial (108–110). The HER2 proto-oncogene is closely related to the occurrence of epithelial ovarian cancer; therefore, it provides an ideal target for the treatment of ovarian cancer. Trastuzumab, pertuzumab, olaparib, and lapatinib are widely approved by the Food and Drug Administration (FDA) for the treatment of HER2-positive cancers. However, clinical studies have shown that HER2-poszitive cancers are prone to become resistant to trastuzumab and pertuzumab; resistance mainly involves crosstalk with heterologous receptors, amplification, and the destruction of binding sites to HER2 receptors (111, 112), which may be related to the abnormal activation of the PI3K-Akt signaling pathway leading to PI3KCA mutation, phosphatase, and tensin homologue loss (113–115). It is estimated that approximately half of patients with HER2-positive metastatic breast cancer do not respond to trastuzumab (116). Lapatinib has shown positive efficacy in patients with HER2-positive ovarian cancer, but its efficacy in patients with ovarian cancer is only 22% to 30% (117, 118). Therefore, many researchers have recently focused on finding new methods and approaches for the comprehensive treatment of ovarian cancer through radioimmunotherapy. To date, many studies have focused on the targeted therapy of a variety of radionuclide-labeled HER2 affibody, including lutetium-177 (^177^Lu), ^64^Cu, and yttrium-90 (^90^Y). Nevertheless, studies have shown that molecular probes of the radionuclide ^64^Cu and ^90^Y-labeled HER2 affibody need to be further optimized due to higher radioactive uptake in kidney or bone marrow and longer retention time (100, 119). ^177^Lu, with a half-life of 6.7 days, can emit β^-^ particles with three energies of 498 keV (79.3%), 380 keV (9.1%), and 176 keV (12.2%). Due to its relatively low particle energy, the normal tissue around the lesion is less damaged, making it a very suitable radionuclide for therapy (120). In 2007, Tolmachev et al. first used CHX-A”-DTPA as a chelating agent to label affibody Z_HER2:342_ with ^177^Lu; the results showed that ^177^Lu-CHX-A”-DTPA-ABD-(Z_HER2:342_)2 could prevent the growth of SKOV3 ovarian cancer cells highly expressing the HER2 receptor, compared with the control group injected with PBS and simultaneously injected with non-labeled affibody, ^177^Lu-CHX-A”-DTPA-ABD-(Z_HER2:342_)2 blocking group. The survival time of nude mice injected with ^177^Lu-CHX-A”-DTPA-ABD-(Z_HER2:342_)2 was significantly prolonged; however, a disadvantage of this affibody is the high radioactive retention in the blood and bone marrow (75). Subsequently, the researchers further linked high-affinity ABD035 to the N-terminus of the affibody Z_HER2:2891_ and linked a DOTA derivative maleimide group to the C-terminus, namely, Z_HER2:2891_-ABD035-DOTA, and then labeled it with ^177^Lu to obtain molecular probe ^177^Lu-ABY-027. This was then compared with that of the previous molecular probe ^177^Lu-CHX-A”-DTPA-ABD-(Z_HER2:342_)2. The results showed that the uptake of the former in the tumor was twice that of the latter, and the radioactive retention in the liver and kidney was significantly reduced (75). To further improve the radioactive uptake of molecular probes by tumors and reduce the radioactive retention of normal organs such as the kidneys, the research team further optimized the molecular probes in a series of studies in which they conducted comparative evaluations *in vivo* and *in vitro*. The results showed that the molecular probe [^177^Lu]Lu-HP16, containing 9 nucleic acid bases, has the highest tumor/kidney uptake ratio and is a promising molecular probe for targeted therapy of HER2-positive ovarian cancer (121–123).

Although there are many advantages that make radionuclide-labeled HER2 affibodies particularly suitable for molecular imaging, many challenges and hurdles remain in the development of these affibodies. One disadvantage is that labeling methods can lead to increased lipophilicity, often leading to off-target interactions with normal tissues and binding to blood proteins (124). Another disadvantage may be the bacterial source of the protein scaffold, as repeated use in patient treatment leads to an increased risk of immunogenicity (125).

## Discussion

HER2 is found in the cytoplasm of normal cells, mainly in the mitochondrial cristae, with little amounts occurring on the cell membrane; HER2 is found only on the cell membrane in tumor cells. The expression of HER2 in normal ovarian epithelium is very low; however, the expression in epithelial ovarian tumor tissue has been reported to be high at 55 times that of normal ovarian epithelium (126). Epithelial ovarian cancer consists of histological subtypes, which include serous carcinoma (prevalence 75%), mucinous carcinoma (20%), endometrioid carcinoma (2%), and clear cell carcinoma (<1%). Regarding HER2 positivity, serous (29%) and mucinous carcinomas (38%) had higher positivity than endometrioid (20%) and clear cell carcinomas (23.1%), while the differential expressions of HER2 among different tissue types in epithelial ovarian carcinoma were important biomarkers for prognosis in epithelial ovarian cancer (127).

Fluorodeoxyglucose (FDG) labeled with radionuclide ^18^F is widely used in clinical practice and has been proved to be highly accurate in the differential diagnosis of benign and malignant ovarian masses (128, 129). However, a study of ^18^F-FDG PET/CT to evaluate the expression status of HER2 receptor has not been reported. Affibody has high affinity for HER2-expressing tumors, low molecular weight, stable biological activity, and fast blood clearance time (130). Recently, the use of molecular probes from radionuclide-labeled affibody has shown promise in tumor molecular imaging research. However, there are many difficulties in the preparation of molecular probes from radionuclide-labeled HER2 affibody that need to be addressed to enable more effective use in clinical practice. Most notably, research needs to focus on the reduction of non-specific uptake in the liver, kidney, bone marrow, and gastrointestinal tract. To this end, researchers have modified the amino acid sequence to change the molecular surface charge, lipid solubility, and affinity, and used different radionuclides to optimize the labeling method, improving the specific uptake of molecular probes at the tumor site, reducing the uptake of non-target organs, thereby optimizing the distribution *in vivo*. Moreover, different chelating agents have been introduced into the radionuclide labeling of HER2 affibody. Increasing the hydrophilicity of the chelating agent can reduce the non-specific uptake of the liver, enable the molecular probe to be excreted through the kidney, reduce the uptake of the gastrointestinal tract, and thus reduce the background of the image, increase the target/non-target ratio, and lead to an improved image. To date, more than 20 chelating agents have been introduced into molecular probes for radionuclide-labeled HER2 affibody. The advantages and disadvantages of commonly used chelators are shown in **Table 2**.

**Table 2:** Overview of advantages and disadvantages of commonly used chelators as molecular probes.

Chelating agent	Full name	Representative molecular probes	Metabolic pathways	Benifits	Disadvantages
-maGSG-	mercaptoacetyl-glycyl-seryl-glycyl	^99m^Tc-maGSG-Z_HER2:342_	kidney	low liver and gastrointestinal uptake	hign renal uptake
-maESE-	mercaptoacetyl-glutamyl-seryl-glutamyl	^99m^Tc-maESE-Z_HER2:342_	liver	high affinity; low image background; low renal uptake	relatively high liver uptake
-PIB-	Iodobenzoate	^125^I-PIB-Z_HER2:342_	liver	high affinity; low renal uptake	untargeted uptake
-HPEM-	4-hydroxyphenyl-ethyl-maleimide	^131^I/^125^I-HPEM-Z_HER2:342_-C	liver	high affinity; low renal uptake	hign liver and gastrointestinal uptake
-DOTA-	1,4,7,l0-tetraazacyclodo-decane-*N,N,N,N*-tetra acetic acid	^111^In-DOTA-Z_HER2:342-pep2_	kidney	high affinity; fast blood clearance; low image background	high renal uptake
		^68^Ga-DOTA-Z_HER2:342min_			
-NOTA-	1,4,7-triazacyclononane-*N,N,N*-triacetic acid	^68^Ga-NOTA-Z_HER2:S1_	kidney	high affinity; low image background	high renal uptake
		^64^Cu-NOTA-Z_HER2:S1_			
-FBO-	(N-(4-flfluorobenzylidene)oxime)	^18^F-FBO-Z_HER2:477_	liver, kidney	high affinity	high liver and renal uptake
-FBEM-	N-[2-(4-fluoro-benzamido)ethyl] maleimide	^18^F-FBEM-Z_HER2:342_	kidney	high affinity; low liver uptake	high renal and bone uptake
-FET-	fluoroethyl-L-tyrosine	[^18^F]FET-Z_HER2:342_	kidney	high affinity; low liver uptake	low yield
-NODAGA-	1-(1,3-carboxypropyl)-4,7-carboxymethyl-1,4,7-triazacyclononane	^68^Ga-NODAGA-Z_HER2:S1_	kidney	high affinity; low image background; low liver uptake	high bone marrow uptake
		^64^Cu-NODAGA-Z_HER2:S1_			
-maGSG-	mercaptoacetyl-glycyl-seryl-glycyl	^186^Re-maGSG-Z_HER2:342_	liver	high affinity; low renal uptake	high liver uptake
		^99m^Tc-maSGS-Z_HER2:342_	kidney	high affinity; low liver uptake	high renal uptake
-maGGG-	mercaptoacetyl-glycyl-glycyl-glycyl	^99m^Tc-maGGG-Z_HER2:342_	not applicable	high affinity; low liver and renal uptake	high image background
		^186^Re-maGGG-Z_HER2:342_			

The median survival of patients with ovarian cancer has been significantly improved recently through the introduction of novel drug treatments and minimally invasive surgical techniques (22). However, the symptomatology, diagnosis, and treatment of ovarian cancer still pose great challenges. Multidisciplinary cooperation and multiple detection methods are needed to provide gynecological oncologists with as much detailed clinical data as possible to optimally design appropriate treatment plans for each patient (22, 131). HER2 is overexpressed or amplified in various malignancies. Therefore, therapeutic drugs that target the linkage of HER2-positive tumor cells to a peptide with a high affinity for the HER2 receptor should be developed to target a variety of HER2-positive malignancies (132). To date, a variety of HER2-targeting therapeutic drugs have been developed, including HER2 addressing therapeutic monoclonal antibodies, nanobodies, and affibodies. Several of these drugs have entered the clinical trial stage, and the results of clinical studies have shown that they benefit patients with HER2-positive breast cancer. However, the study and use of radionuclide-labeled HER2 affibody have been hampered by its short residence time in the blood (133).

In conclusion, the molecular probe of radionuclide-labeled HER2 affibody can accurately evaluate the expression status of HER2 receptor in epithelial ovarian cancer, which provides favorable conditions for the localization, diagnosis of cancer foci, targeted therapy, and prognosis evaluation. The research and development of new molecular probes for nuclear medicine aims to introduce radionuclides into cancer foci in a targeted manner, display systemic lesions, and kill tumor cells through radionuclides’ radioactivity. Continued progress will result in improved methods for the integration of the diagnosis and treatment of ovarian cancer. Nuclear medicine imaging technology is an important imaging method for basic and clinical research on malignant tumors. A variety of radionuclide-labeled HER2 affibody molecular probes have been successfully developed and have shown potential in preclinical experimental studies of ovarian cancer, laying a foundation for multi-center clinical trials. In the future, the research and development of HER2 affibody molecular probes will focus on optimizing the labeling method of radionuclides, using different chelating agents, or modifying the amino acid sequence to further improve the specific uptake of molecular probes in tumor sites, while reducing the uptake of non-target organs, especially in the liver, kidney, bone marrow, and gastrointestinal tract. This will optimize the distribution in the body, and the radionuclide-labeled HER2 small-molecule targeting binding protein can then be used in clinical practice as soon as possible to enable tumor-specific diagnosis, and to promote the evaluation of the therapeutic efficacy of targeted therapy drugs objectively at an early stage.

## Author Contributions

XH compiled/interpreted resources and was the primary author for majority of the manuscript. DL assisted with manuscript writing. YF assisted with manuscript edits. JZ and ZF developed the conception and overall design of the project/paper. JC and PW provided manuscript editing/revisions and interpretation of resources to include in the paper. All authors contributed to manuscript revision, and read and approved the submitted version.

## Funding

This study was funded by the National Natural Science Foundation of the People’s Republic of China, NSFC (grant number: 81571712), Zunyi Medical College Research Start Fund 2018ZYFY03, and QianKeHe platform talents [2017] (grant number: 5733-035).

## Conflict of Interest

Author YF was employed by Jiangsu Yuanben Biotechnology Co., Ltd.

The remaining authors declare that the research was conducted in the absence of any commercial or financial relationships that could be construed as a potential conflict of interest.

## Publisher’s Note

All claims expressed in this article are solely those of the authors and do not necessarily represent those of their affiliated organizations, or those of the publisher, the editors and the reviewers. Any product that may be evaluated in this article, or claim that may be made by its manufacturer, is not guaranteed or endorsed by the publisher.

## References

[1] Sung H Ferlay J Siegel RL Laversanne M Soerjomataram I Jemal A. Global Cancer Statistics 2020: GLOBOCAN Estimates of Incidence and Mortality Worldwide for 36 Cancers in 185 Countries. CA Cancer J Clin (2021) 71(3):209–49. doi: 10.3322/caac.21660 33538338

[2] Wang B Liu SZ Zheng RS Zhang F Chen WQ Sun XB. Time Trends of Ovarian Cancer Incidence in China. Asian Pac J Cancer Prev (2014) 15(1):191–3. doi: 10.7314/APJCP.2014.15.1.191 24528025

[3] Huang Z Zheng Y Wen W Wu C Bao P Wang C. Incidence and Mortality of Gynaecological Cancers: Secular Trends in Urban Shanghai, China Over 40 Years. Eur J Cancer (2016) 63:1–10. doi: 10.1016/j.ejca.2016.04.016 27254837 PMC4942399

[4] Westhoff C. Ovarian Cancer. Annu Rev Publie Health (1996) 17:85–96. doi: 10.1146/annurev.pu.17.050196.000505 8724217

[5] Hoekstra A Rodriguez GC. Chemoprevention of Ovarian Cancer. Cancer Treat Res (2009) 149:3–34. doi: 10.1007/978-0-387-98094-2_1 19763429

[6] Hanna L Adams M. Prevention of Ovarian Cancer. Best Pract Res Clin Obstet Gynaecol (2006) 20:339–62. doi: 10.1016/j.bpobgyn.2005.10.016 16368271

[7] Shan W Liu J. Inflammation: A Hidden Path to Breaking the Spell of Ovarian Cancer. Cell Cycle (2009) 8:3107–11. doi: 10.4161/cc.8.19.9590 19738436

[8] Toriola AT Grankvist K Agborsangaya CB Lukanova A Lehtinen M Surcel HM. Changes in Pre-Diagnostic Serum C-Reactive Protein Concentrations and Ovarian Cancer Risk: A Longitudinal Study. Ann Oncol (2011) 22(8):1916–21. doi: 10.1093/annonc/mdq694 21292643

[9] Lin HW Tu YY Lin SY Su WJ Lin WL Lin WZ. Risk of Ovarian Cancer in Women With Pelvic Inflammatory Disease: A Population-Based Study. Lancet Oncol (2011) 12(9):900–4. doi: 10.1016/S1470-2045(11)70165-6 21835693

[10] Chittenden BG Fullerton G Maheshwari A Bhattacharya S. Polycystic Ovary Syndrome and the Risk of Gynaecological Cancer: A Systematic Review. Reprod BioMed Online (2009) 19(3):398–405. doi: 10.1016/S1472-6483(10)60175-7 19778486

[11] Al-Sabbagh M Lam EW Brosens JJ. Mechanisms of Endometrial Progesterone Resistance. Mol Cell Endocrinol (2012) 358(2):208–15. doi: 10.1016/j.mce.2011.10.035 22085558

[12] Whiteman DC Siskind V Purdie DM Green AC. Timing of Pregnancy and the Risk of Epithelial Ovarian Cancer. Cancer Epidemiol Biomarkers Prev (2003) 12(1):42–6. 12540502

[13] Collaborative Group on Epidemiological Studies of Ovarian Cancer Beral V Doll R Hermon C Peto R Reeves G. Ovarian Cancer and Oral Contraceptives: Collaborative Reanalysis of Data From 45 Epidemiological Studies Including 23,257 Women With Ovarian Cancer and 87,303 Controls. Lancet (2008) 371(9609):303–14. doi: 10.1016/S0140-6736(08)60167-1 18294997

[14] Danforth KN Tworoger SS Hecht JL Rosner BA Colditz GA Hankinson SE. Breastfeeding and Risk of Ovarian Cancer in Two Prospective Cohorts. Cancer Causes Control (2007) 18(5):517–23. doi: 10.1007/s10552-007-0130-2 17450440

[15] Terry KL Schock H Fortner RT Hüsing A Fichorova RN Yamamoto HS. A Prospective Evaluation of Early Detection Biomarkers for Ovarian Cancer in the European EPIC Cohort. Clin Cancer Res (2016) 22(18):4664–75. doi: 10.1158/1078-0432.CCR-16-0316 PMC5026545 27060155

[16] Mathieu KB Bedi DG Thrower SL Qayyum A Bast RC Jr.. Screening for Ovarian Cancer: Imaging Challenges and Opportunities for Improvement. Ultrasound Obstet Gynecol (2018) 51(3):293–303. doi: 10.1002/uog.17557 28639753 PMC5788737

[17] Schechter AL Stern DF Vaidyanathan L Decker SJ Drebin JA Greene MI. The Neu Oncogene: An Erb-B-Related Gene Encoding a 185,000-Mr Tumour Antigen. Nature (1984) 312(5994):513–6. doi: 10.1038/312513a0 6095109

[18] Connell CM Doherty GJ. Activating HER2 Mutations as Emerging Targets in Multiple Solid Cancers. ESMO Open (2017) 2(5):e000279. doi: 10.1136/esmoopen-2017-000279 29209536 PMC5708307

[19] Lee ES Lee Y Suh D Kang J Kim I. Detection of HER-2 and EGFR Gene Amplification Using Chromogenic in-Situ Hybridization Technique in Ovarian Tumors. Appl Immunohistochem Mol Morphol (2010) 18(1):69–74. doi: 10.1097/PAI.0b013e3181af7d3f 19652593

[20] Corkery DP Le Page C Meunier L Provencher D Mes-Masson AM Dellaire G. PRP4K Is a HER2-Regulated Modifier of Taxane Sensitivity. Cell Cycle (2015) 14(7):1059–69. doi: 10.1080/15384101.2015.1007775 PMC4612451 25602630

[21] Cloven NG Kyshtoobayeva A Burger RA Yu IR Fruehauf JP. *In Vitro* Chemoresistance and Biomarker Profiles are Unique for Histologic Subtypes of Epithelial Ovarian Cancer. Gynecol Oncol (2004) 92(1):160–6. doi: 10.1016/j.ygyno.2003.09.030 14751152

[22] Falzone L Scandurra G Lombardo V Gattuso G Lavoro A Distefano AB. A Multidisciplinary Approach Remains the Best Strategy to Improve and Strengthen the Management of Ovarian Cancer (Review). Int J Oncol (2021) 59(1):53. doi: 10.3892/ijo.2021.5233 34132354 PMC8208622

[23] Orr B Edwards RP. Diagnosis and Treatment of Ovarian Cancer. Hematol Oncol Clin North Am (2018) 32(6):943–64. doi: 10.1016/j.hoc.2018.07.010 30390767

[24] Dilley J Burnell M Gentry-Maharaj A Ryan A Neophytou C Apostolidou S. Ovarian Cancer Symptoms, Routes to Diagnosis and Survival - Population Cohort Study in the ‘No Screen’ Arm of the UK Collaborative Trial of Ovarian Cancer Screening (UKCTOCS). Gynecol Oncol (2020) 158(2):316–22. doi: 10.1016/j.ygyno.2020.05.002 PMC7453382 32561125

[25] Moelans CB de Weger RA van der Wall E van Diest PJ. Current Technologies for HER2 Testing in Breast Cancer. Crit Rev Oncol Hematol (2011) 80(3):380–92. doi: 10.1016/j.critrevonc.2010.12.005 21273092

[26] Schlüter B Gerhards R Strumberg D Voigtmann R. Combined Detection of Her2/neu Gene Amplification and Protein Overexpression in Effusions From Patients With Breast and Ovarian Cancer. J Cancer Res Clin Oncol (2010) 136(9):1389–400. doi: 10.1007/s00432-010-0790-2 PMC11827919 20217132

[27] Jiang D Im HJ Sun H Valdovinos HF England CG Ehlerding EB. Radiolabeled Pertuzumab for Imaging of Human Epidermal Growth Factor Receptor 2 Expression in Ovarian Cancer. Eur J Nucl Med Mol Imaging (2017) 44(8):1296–305. doi: 10.1007/s00259-017-3663-y PMC5471126 28265738

[28] Schlüter B Gerhards R Strumberg D Voigtmann R. Combined Detection of Her2/neu Gene Amplification and Protein Overexpression in Effusions From Patients With Breast and Ovarian Cancer. J Cancer Res Clin Oncol (2010) 136(9):1389–400. doi: 10.1007/s00432-010-0790-2 PMC11827919 20217132

[29] Hillig T Thode J Breinholt MF Franzmann MB Pedersen C Lund F. Assessing HER2 Amplification by IHC, FISH, and Real-Time Polymerase Chain Reaction Analysis (Real-Time PCR) Following LCM in Formalin-Fixed Paraffin Embedded Tissue From 40 Women With Ovarian Cancer. APMIS (2012) 120(12):1000–7. doi: 10.1111/j.1600-0463.2012.02929.x PMC3533780 23030524

[30] Nilsson B Moks T Jansson B Abrahmsén L Elmblad A Holmgren E. A Synthetic IgG-Binding Domain Based on Staphylococcal Protein a. Protein Eng (1987) 1(2):107–13. doi: 10.1093/protein/1.2.107 3507693

[31] Braisted AC Wells JA. Minimizing a Binding Domain From Protein a. Proc Natl Acad Sci USA (1996) 93(12):5688–92. doi: 10.1073/pnas.93.12.5688 PMC39121 8650153

[32] Nord K Nilsson J Nilsson B Uhlén M Nygren PA. A Combinatorial Library of an Alpha-Helical Bacterial Receptor Domain. Protein Eng (1995) 8(6):601–8. doi: 10.1093/protein/8.6.601 8532685

[33] Löfblom J Feldwisch J Tolmachev V Carlsson J Ståhl S Frejd FY. Affibody Molecules: Engineered Proteins for Therapeutic, Diagnostic and Biotechnological Applications. FEBS Lett (2010) 584(12):2670–80. doi: 10.1016/j.febslet.2010.04.014 20388508

[34] Nord K Gunneriusson E Ringdahl J Ståhl S Uhlén M Nygren PA. Binding Proteins Selected From Combinatorial Libraries of an Alpha-Helical Bacterial Receptor Domain. Nat Biotechnol (1997) 15(8):772–7. doi: 10.1038/nbt0897-772 9255793

[35] Nygren PA. Alternative Binding Proteins: Affibody Binding Proteins Developed From a Small Three-Helix Bundle Scaffold. FEBS J (2008) 275(11):2668–76. doi: 10.1111/j.1742-4658.2008.06438.x 18435759

[36] Jamous M Haberkorn U Mier W. Synthesis of Peptide Radiopharmaceuticals for the Therapy and Diagnosis of Tumor Diseases. Molecules (2013) 18(3):3379–409. doi: 10.3390/molecules18033379 PMC6269889 23493103

[37] Weidle UH Auer J Brinkmann U Georges G Tiefenthaler G. The Emerging Role of New Protein Scaffold-Based Agents for Treatment of Cancer. Cancer Genomics Proteomics (2013) 10(4):155–68. 23893924

[38] Antunes P Ginj M Walter MA. Influence of Different Spacers on the Biological Profile of a DOTA-Somatostatin Analogue. Bioconjug Chem (2007) 18(1):84–92. doi: 10.1021/bc0601673 17226960

[39] Ståhl S Gräslund T Eriksson Karlström A Frejd FY Nygren PÅ Löfblom J. Affibody Molecules in Biotechnological and Medical Applications. Trends Biotechnol (2017) 35(8):691–712. doi: 10.1016/j.tibtech.2017.04.007 28514998

[40] Baum RP Prasad V Müller D Schuchardt C Orlova A Wennborg A. Molecular Imaging of HER2-Expressing Malignant Tumors in Breast Cancer Patients Using Synthetic 111In or 68Ga 1abeled Affibody Molecules. J Nucl Med (2010) 51(6):892–97. doi: 10.2967/jnumed.109.073239 20484419

[41] Sörensen J Sandberg D Sandström M Wennborg A Feldwisch J Tolmachev V. First-In-Human Molecular Imaging of HER2 Expression in Breast Cancer Metastases Using the 111In-ABY-025 Affibody Molecule. J Nucl Med (2014) 55(5):730–5. doi: 10.2967/jnumed.113.131243 24665085

[42] Steffen AC Wikman M Tolmachev V Adams GP Nilsson FY Ståhl S. *In Vitro* Characterization of a Bivalent Anti-HER-2 Affibody With Potential for Radionuclide-Based Diagnostics. Cancer Biother Radiopharm (2005) 20(3):239–48. doi: 10.1089/cbr.2005.20.239 15989469

[43] Steffen AC Orlova A Wikman M Nilsson FY Ståhl S Adams GP. Affibody-Mediated Tumour Targeting of HER-2 Expressing Xenografts in Mice. Eur J Nucl Med Mol Imaging (2006) 33(6):631–8. doi: 10.1007/s00259-005-0012-3 16538504

[44] Orlova A Magnusson M Eriksson TL Nilsson M Larsson B Höidén-Guthenberg I. Tumor Imaging Using a Picomolar Affinity HER2 Binding Affibody Molecule. Cancer Res (2006) 66(8):4339–48. doi: 10.1158/0008-5472.CAN-05-3521 16618759

[45] Ahlgren S Orlova A Rosik D Sandström M Sjöberg A Baastrup B. Evaluation of Maleimide Derivative of DOTA for Site-Specific Labeling of Recombinant Affibody Molecules. Bioconjug Chem (2008) 19(1):235–43. doi: 10.1021/bc700307y 18163536

[46] Altai M Perols A Karlström AE Sandström M Boschetti F Orlova A. Preclinical Evaluation of Anti-HER2 Affibody Molecules Site-Specifically Labeled With 111In Using a Maleimido Derivative of NODAGA. Nucl Med Biol (2012) 39(4):518–29. doi: 10.1016/j.nucmedbio.2011.10.013 22172396

[47] Oroujeni M Rinne SS Vorobyeva A Loftenius A Feldwisch J Jonasson P. Preclinical Evaluation of 99mtc-ZHER2:41071, a Second-Generation Affibody-Based HER2-Visualizing Imaging Probe With a Low Renal Uptake. Int J Mol Sci (2021) 22(5):2770. doi: 10.3390/ijms22052770 33803361 PMC7967187

[48] Tolmachev V Velikyan I Sandström M Orlova A. A HER2-Binding Affibody Molecule Labelled With 68Ga for PET Imaging: Direct *In Vivo* Comparison With the 111In-Labelled Analogue. Eur J Nucl Med Mol Imaging (2010) 37(7):1356–67. doi: 10.1007/s00259-009-1367-7 20130858

[49] Wållberg H Orlova A. Slow Internalization of Anti-HER2 Synthetic Affibody Monomer 111In-DOTA-ZHER2:342-Pep2: Implications for Development of Labeled Tracers. Cancer Biother Radiopharm (2008) 23(4):435–42. doi: 10.1089/cbr.2008.0464 18771347

[50] Tolmachev V Nilsson FY Widström C Andersson K Rosik D Gedda L. 111In-Benzyl-DTPA-ZHER2:342, an Affibody-Based Conjugate for *In Vivo* Imaging of HER2 Expression in Malignant Tumors. J Nucl Med (2006) 47(5):846–53. 16644755

[51] Rosik D Orlova A Malmberg J Altai M Varasteh Z Sandström M. Direct Comparison of 111In-Labelled Two-Helix and Three-Helix Affibody Molecules for *In Vivo* Molecular Imaging. Eur J Nucl Med Mol Imaging (2012) 39(4):693–702. doi: 10.1007/s00259-011-2016-5 22170322

[52] Orlova A Rosik D Sandström M Lundqvist H Einarsson L Tolmachev V. Evaluation of [(111/114m)In]CHX-A”-DTPA-ZHER2:342, an Affibody Ligand Coniugate for Targeting of HER2-Expressing Malignant Tumors. Q J Nucl Med Mol Imaging (2007) 51(4):314–23. 17464277

[53] Baum RP Prasad V Muller D Schuchardt C Orlova A Wennborg A. Molecular Imaging of HER2-Expressing Malignant Tumors in Breast Cancer Patients Using Synthetic 111In- or 68Ga-Labeled Affibody Molecules. J Nucl Med (2010) 51(6):892–7. doi: 10.2967/jnumed.109.073239 20484419

[54] Altai M Perols A Karlström AE Sandström M Boschetti F Orlova A. Preclinical Evaluation of Anti-HER2 Affibody Molecules Site-Specifically Labeled With 111In Using a Maleimido Derivative of NODAGA. Nucl Med Biol (2012) 39(4):518–29. doi: 10.1016/j.nucmedbio.2011.10.013 22172396

[55] Engfeldt T Orlova A Tran T Bruskin A Widström C Karlström AE. Imaging of HER2-Expressing Tumours Using a Synthetic Affibody Molecule Containing the 99mtc-Chelating Mercaptoacetyl-Glycyl-Glycyl-Glycyl (MAG3) Sequence. Eur J Nucl Med Mol Imaging (2007) 34(5):722–33. doi: 10.1007/s00259-006-0266-4 17146656

[56] Tran T Engfeldt T Orlova A Sandström M Feldwisch J Abrahmsén L. (99m)Tc-maEEE-Z(HER2:342), an Affibody Molecule-Based Tracer for the Detection of HER2 Expression in Malignant Tumors. Bioconjug Chem (2007) 18(6):1956–64. doi: 10.1021/bc7002617 17944527

[57] Engfeldt T Tran T Orlova A Widström C Feldwisch J Abrahmsen L. 99mtc-Chelator Engineering to Improve Tumour Targeting Properties of a HER2-Specific Affibody Molecule. Eur J Nucl Med Mol Imaging (2007) 34(11):1843–53. doi: 10.1007/s00259-007-0474-6 17565496

[58] Ekblad T Tran T Orlova A Widström C Feldwisch J Abrahmsén L. Development and Preclinical Characterisation of 99mtc-Labelled Affibody Molecules With Reduced Renal Uptake. Eur J Nucl Med Mol Imaging (2008) 35(12):2245–55. doi: 10.1007/s00259-008-0845-7 18594815

[59] Tran TA Ekblad T Orlova A Sandström M Feldwisch J Wennborg A. Effects of Lysine-Containing Mercaptoacetyl-Based Chelators on the Biodistribution of 99mtc-Labeled Anti-HER2 Affibody Molecules. Bioconjug Chem (2008) 19(12):2568–76. doi: 10.1021/bc800244b 19035668

[60] Han J Zhao Y Zhao X Ma T Hao T Liu J. Therapeutic Efficacy and Imaging Assessment of the HER2-Targeting Chemotherapy Drug ZHER2:V2-Pemetrexed in Lung Adenocarcinoma Xenografts. Invest New Drugs (2020) 38(4):1031–43. doi: 10.1007/s10637-019-00876-3 31758360

[61] Jiao H Zhao X Liu J Ma T Zhang Z Zhang J. *In Vivo* Imaging Characterization and Anticancer Efficacy of a Novel HER2 Affibody and Pemetrexed Conjugate in Lung Cancer Model. Nucl Med Biol (2019) 68-69:31–9. doi: 10.1016/j.nucmedbio.2018.11.004 30578135

[62] Wållberg H Orlova A Altai M Hosseinimehr SJ Widström C Malmberg J. Molecular Design and Optimization of 99mtc-Labeled Recombinant Affibody Molecules Improves Their Biodistribution and Imaging Properties. J Nucl Med (2011) 52(3):461–9. doi: 10.2967/jnumed.110.083592 21321280

[63] Yang Y Zhao X Xing Y Yu T Zhang J Wang J. Preclinical Evaluation of 99mtc Direct Labeling ZHER2:V2 for HER2 Positive Tumors Imaging. Oncol Lett (2018) 16(4):5361–66. doi: 10.3892/ol.2018.9279 PMC6144291 30250607

[64] Cai J Li X Mao F Wang P Luo Y Zheng K. Non-Invasive Monitoring of HER2 Expression in Breast Cancer Patients With 99mtc-Affibody SPECT/Ct. Iran J Radiol (2020) 17(1):e96419. doi: 10.5812/iranjradiol.96419

[65] Strand J Nordeman P Honarvar H Altai M Orlova A Larhed M. Site-Specific Radioiodination of HER2-Targeting Affibody Molecules Using 4-Iodophenethylmaleimide Decreases Renal Uptake of Radioactivity. ChemistryOpen (2015) 4(2):174–82. doi: 10.1002/open.201402097 PMC4420590 25969816

[66] Kramer-Marek G Bernardo M Kiesewetter DO Bagci U Kuban M Aras O. PET of HER2-Positive Pulmonary Metastases With 18F-ZHER2:342 Affibody in a Murine Model of Breast Cancer: Comparison With 18F-FDG. J Nucl Med (2012) 53(6):939–46. doi: 10.2967/jnumed.111.100354 PMC7497802 22582046

[67] Kramer-Marek G Kiesewetter DO Martiniova L Jagoda E Lee SB Capala J. [18f]FBEM-Z(HER2:342)-Affibody Molecule-a New Molecular Tracer for *In Vivo* Monitoring of HER2 Expression by Positron Emission Tomography. Eur J Nucl Med Mol Imaging (2008) 35(5):1008–18. doi: 10.1007/s00259-007-0658-0 PMC2365742 18157531

[68] Yanai A Harada R Iwata R Yoshikawa T Ishikawa Y Furumoto S. Site-Specific Labeling of F-18 Proteins Using a Supplemented Cell-Free Protein Synthesis System and O-2-[18f]Fluoroethyl-L-Tyrosine: [18f]FET-HER2 Affibody Molecule. Mol Imaging Biol (2019) 21(3):529–37. doi: 10.1007/s11307-018-1266-z 30112727

[69] Ren G Zhang R Liu Z Webster JM Miao Z Gambhir SS. A 2-Helix Small Protein Labeled With 68Ga for PET Imaging of HER2 Expression. J Nucl Med (2009) 50(9):1492–9. doi: 10.2967/jnumed.109.064287 PMC4216181 19690041

[70] Sandstrom M Lindskog K Velikyan I Wennborg A Feldwisch J Sandberg D. Biodistribution and Radiation Dosimetry of the Anti-HER2 Affibody Molecule 68Ga-ABY-025 in Breast Cancer Patients. J Nucl Med (2016) 57(6):867–71. doi: 10.2967/jnumed.115.169342 26912439

[71] Sorensen J Velikyan I Sandberg D Wennborg A Feldwisch J Tolmachev V. Measuring HER2-Receptor Expression in Metastatic Breast Cancer Using [68Ga]ABY-025 Affibody PET/Ct. Theranostics (2016) 6(2):262–71. doi: 10.7150/thno.13502 PMC4729774 26877784

[72] Qi S Hoppmann S Xu Y Cheng Z. PET Imaging of HER2-Positive Tumors With Cu-64-Labeled Affibody Molecules. Mol Imaging Biol (2019) 21(5):907–16. doi: 10.1007/s11307-018-01310-5 30617730

[73] Altai M Wållberg H Honarvar H Strand J Orlova A Varasteh Z. 188Re-ZHER2:V2, a Promising Affibody-Based Targeting Agent Against HER2-Expressing Tumors: Preclinical Assessment. J Nucl Med (2014) 55(11):1842–8. doi: 10.2967/jnumed.114.140194 25278516

[74] Altai M Honarvar H Wållberg H Strand J Varasteh Z Rosestedt M. Selection of an Optimal Cysteine-Containing Peptide-Based Chelator for Labeling of Affibody Molecules With (188)Re. Eur J Med Chem (2014) 87:519–28. doi: 10.1016/j.ejmech.2014.09.082 25282673

[75] Tolmachev V Orlova A Pehrson R Galli J Baastrup B Andersson K. Radionuclide Therapy of HER2-Positive Microxenografts Using a 177Lu-Labeled HER2-Specific Affibody Molecule. Cancer Res (2007) 67(6):2773–82. doi: 10.1158/0008-5472.CAN-06-1630 17363599

[76] Honarvar H Westerlund K Altai M Sandström M Orlova A Tolmachev V. Feasibility of Affibody Molecule-Based PNA-Mediated Radionuclide Pretargeting of Malignant Tumors. Theranostics (2016) 6(1):93–103. doi: 10.7150/thno.12766 26722376 PMC4679357

[77] Ahlgren S Orlova A Wållberg H Hansson M Sandström M Lewsley R. Targeting of HER2-Expressing Tumors Using 111In-ABY-025, a Second-Generation Affibody Molecule With a Fundamentally Reengineered Scaffold. J Nucl Med (2010) 51(7):1131–8. doi: 10.2967/jnumed.109.073346 20554729

[78] Heskamp S Laverman P Rosik D Boschetti F van der Graaf WT Oyen WJ. Imaging of Human Epidermal Growth Factor Receptor Type 2 Expression With 18F-Labeled Affibody Molecule ZHER2:2395 in a Mouse Model for Ovarian Cancer. J Nucl Med (2012) 53(1):146–53. doi: 10.2967/jnumed.111.093047 22173842

[79] Tolmachev V Xu H Wållberg H Ahlgren S Hjertman M Sjöberg A. Evaluation of a Maleimido Derivative of CHX-A” DTPA for Site-Specific Labeling of Affibody Molecules. Bioconjug Chem (2008) 19(8):1579–87. doi: 10.1021/bc800110y PMC2561312 18620447

[80] Altai M Varasteh Z Andersson K Eek A Boerman O Orlova A. *In Vivo* and *In Vitro* Studies on Renal Uptake of Radiolabeled Affibody Molecules for Imaging of HER2 Expression in Tumors. Cancer Biother Radiopharm (2013) 28(3):187–95. doi: 10.1089/cbr.2012.1304 23461385

[81] Westerlund K Honarvar H Norrström E Strand J Mitran B Orlova A. Increasing the Net Negative Charge by Replacement of DOTA Chelator With DOTAGA Improves the Biodistribution of Radiolabeled Second-Generation Synthetic Affibody Molecules. Mol Pharm (2016) 13(5):1668–78. doi: 10.1021/acs.molpharmaceut.6b00089 27010700

[82] Lindberg H Hofström C Altai M Honorvar H Wållberg H Orlova A. Evaluation of a HER2-Targeting Affibody Molecule Combining an N-Terminal HEHEHE-Tag With a GGGC Chelator for 99mtc-Labelling at the C Terminus. Tumour Biol (2012) 33(3):641–51. doi: 10.1007/s13277-011-0305-z 22249974

[83] Wållberg H Orlova A Altai M Hosseinimehr SJ Widström C Malmberg J. Molecular Design and Optimization of 99mtc-Labeled Recombinant Affibody Molecules Improves Their Biodistribution and Imaging Properties. J Nucl Med (2011) 52(3):461–9. doi: 10.2967/jnumed.110.083592 21321280

[84] Yang Y Zhao X Xing Y Yu T Zhang J Wang J. Preclinical Evaluation of 99mtc Direct Labeling ZHER2:V2 for HER2 Positive Tumors Imaging. Oncol Lett (2018) 16(4):5361–6. doi: 10.3892/ol.2018.9279 PMC6144291 30250607

[85] Strand J Nordeman P Honarvar H Altai M Orlova A Larhed M. Site-Specific Radioiodination of HER2-Targeting Affibody Molecules Using 4-Iodophenethylmaleimide Decreases Renal Uptake of Radioactivity. ChemistryOpen (2015) 4(2):174–82. doi: 10.1002/open.201402097 PMC4420590 25969816

[86] Steffen AC Orlova A Wikman M Nilsson FY Ståhl S Adams GP. Affibody-Mediated Tumour Targeting of HER-2 Expressing Xenografts in Mice. Eur J Nucl Med Mol Imaging (2006) 33(6):631–8. doi: 10.1007/s00259-005-0012-3 16538504

[87] Tran T Orlova A Sivaev I Sandström M Tolmachev V. Comparison of Benzoate- and Dodecaborate-Based Linkers for Attachment of Radioiodine to HER2-Targeting Affibody Ligand. Int J Mol Med (2007) 19(3):485–93. doi: 10.3892/ijmm.19.3.485 17273798

[88] Tolmachev V Mume E Sjöberg S Frejd FY Orlova A. Influence of Valency and Labelling Chemistry on *In Vivo* Targeting Using Radioiodinated HER2-Binding Affibody Molecules. Eur J Nucl Med Mol Imaging (2009) 36(4):692–701. doi: 10.1007/s00259-008-1003-y 19066886

[89] Vorobyeva A Schulga A Rinne SS Günther T Orlova A Deyev S. Indirect Radioiodination of DARPin G3 Using N-Succinimidyl-Para-Iodobenzoate Improves the Contrast of HER2 Molecular Imaging. Int J Mol Sci (2019) 20(12):3047. doi: 10.3390/ijms20123047 PMC6627094 31234471

[90] Beyer T Townsend DW Brun T Kinahan PE Charron M Roddy R. A Combined PET/CT Scanner for Clinical Oncology. J Nucl Med (2000) 41(8):1369–79. 10945530

[91] Vahidfar N Farzanefar S Ahmadzadehfar H Molloy EN Eppard E. A Review of Nuclear Medicine Approaches in the Diagnosis and the Treatment of Gynecological Malignancies. Cancers (Basel) (2022) 14(7):1779. doi: 10.3390/cancers14071779 35406552 PMC8997132

[92] Altai M Strand J Rosik D Selvaraju RK Eriksson Karlström A. Influence of Nuclides and Chelators on Imaging Using Affibody Molecules: Comparative Evaluation of Recombinant Affibody Molecules Site-Specifically Labeled With ⁶⁸Ga and ¹¹¹In *via* Maleimido Derivatives of DOTA and NODAGA. Bioconjug Chem (2013) 24(6):1102–9. doi: 10.1021/bc300678y 23705574

[93] Strand J Honarvar H Perols A Orlova A Selvaraju RK Karlström AE. Influence of Macrocyclic Chelators on the Targeting Properties of (68)Ga-Labeled Synthetic Affibody Molecules: Comparison With (111)In-Labeled Counterparts. PloS One (2013) 8(8):e70028. 23936372 10.1371/journal.pone.0070028 PMC3731330

[94] Cheng Z De Jesus OP Namavari M De A Levi J Webster JM. Small-Animal PET Imaging of Human Epidermal Growth Factor Receptor Type 2 Expression With Site-Specific 18F-Labeled Protein Scaffold Molecules. J Nucl Med (2008) 49(5):804–13. doi: 10.2967/jnumed.107.047381 PMC4154808 18413392

[95] Iveson PB Glaser M Indrevoll B Shales J Mantzilas D Omtvedt L. FASTlab Radiosynthesis of the 18F-Labelled HER2-Binding Affibody Molecule [18F]GE-226. J Labelled Comp Radiopharm (2019) 62(14):925–32. doi: 10.1002/jlcr.3789 31339589

[96] Trousil S Hoppmann S Nguyen QD Kaliszczak M Tomasi G Iveson P. Positron Emission Tomography Imaging With 18F-Labeled ZHER2:2891 Affibody for Detection of HER2 Expression and Pharmacodynamic Response to HER2-Modulating Therapies. Clin Cancer Res (2014) 20(6):1632–43. doi: 10.1158/1078-0432.CCR-13-2421 24493830

[97] Heskamp S Laverman P Rosik D Boschetti F van der Graaf WT Oyen WJ. Imaging of Human Epidermal Growth Factor Receptor Type 2 Expression With 18F-Labeled Affibody Molecule ZHER2:2395 in a Mouse Model for Ovarian Cancer. J Nucl Med (2012) 53(1):146–53. doi: 10.2967/jnumed.111.093047 22173842

[98] Zinn KR Chaudhuri TR Cheng TP Morris JS Meyer WA Jr.. Production of No-Carrier-Added 64Cu From Zinc Metal Irradiated Under Boron Shielding. Cancer (1994) 73(3 Suppl):774–8. doi: 10.1002/1097-0142(19940201)73:3+<774::AID-CNCR2820731305>3.0.CO;2-L 8306259

[99] Kim JY Park H Lee JC Kim KM Lee KC Ha HJ. A Simple Cu-64 Production and Its Application of Cu-64 ATSM. Appl Radiat Isot (2009) 67(7-8):1190–4. doi: 10.1016/j.apradiso.2009.02.060 19299153

[100] Cheng Z De Jesus OP Kramer DJ De A Webster JM Gheysens O. 64Cu-Labeled Affibody Molecules for Imaging of HER2 Expressing Tumors. Mol Imaging Biol (2010) 12(3):316–24. doi: 10.1007/s11307-009-0256-6 PMC4155984 19779897

[101] Tolmachev V Yim CB Rajander J Perols A Karlström AE Haaparanta-Solin M. Comparative Evaluation of Anti-HER2 Affibody Molecules Labeled With 64Cu Using NOTA and NODAGA. Contrast Media Mol Imaging (2017) 2017:8565802. doi: 10.1155/2017/8565802 29097939 PMC5612711

[102] Orlova A Tran TA Ekblad T Karlström AE Tolmachev V. (186)Re-maSGS-Z (HER2:342), a Potential Affibody Conjugate for Systemic Therapy of HER2-Expressing Tumours. Eur J Nucl Med Mol Imaging (2010) 37(2):260–9. doi: 10.1007/s00259-009-1268-9 19771426

[103] Steffen AC Almqvist Y Chyan MK Lundqvist H Tolmachev V Wilbur DS. Biodistribution of 211At Labeled HER-2 Binding Affibody Molecules in Mice. Oncol Rep (2007) 17(5):1141–7. doi: 10.3892/or.17.5.1141 17390057

[104] Honarvar H Müller C Cohrs S Haller S Westerlund K Karlström AE. Evaluation of the First 44Sc-Labeled Affibody Molecule for Imaging of HER2-Expressing Tumors. Nucl Med Biol (2017) 45:15–21. doi: 10.1016/j.nucmedbio.2016.10.004 27837664

[105] Xu Y Wang L Pan D Yan J Wang X Yang R. Synthesis of a Novel 89Zr-Labeled HER2 Affibody and Its Application Study in Tumor PET Imaging. EJNMMI Res (2020) 10(1):58. doi: 10.1186/s13550-020-00649-7 32495181 PMC7271293

[106] Wållberg H Grafström J Cheng Q Lu L Martinsson Ahlzén HS Samén E. HER2-Positive Tumors Imaged Within 1 Hour Using a Site-Specifically 11C-Labeled Sel-Tagged Affibody Molecule. J Nucl Med (2012) 53(9):1446–53. doi: 10.2967/jnumed.111.102194 22872744

[107] Puglisi F Minisini AM De Angelis C Arpino G. Overcoming Treatment Resistance in HER2-Positive Breast Cancer: Potential Strategies. Drugs (2012) 72(9):1175–93. doi: 10.2165/11634000-000000000-00000 22686613

[108] Loizzi V Ranieri G Laforgia M Gadaleta CD Gargano G Kardhashi A. PARP Inhibitors and Epithelial Ovarian Cancer: Molecular Mechanisms, Clinical Development and Future Prospective. Oncol Lett (2020) 20(4):90. doi: 10.3892/ol.2020.11951 32831909 PMC7439101

[109] Lavoro A Scalisi A Candido S Zanghì GN Rizzo R Gattuso G. Identification of the Most Common BRCA Alterations Through Analysis of Germline Mutation Databases: Is Droplet Digital PCR an Additional Strategy for the Assessment of Such Alterations in Breast and Ovarian Cancer Families? Int J Oncol (2022) 60(5):58. doi: 10.3892/ijo.2022.5349 35383859 PMC8997337

[110] Boussios S Karathanasi A Cooke D Neille C Sadauskaite A Moschetta M. PARP Inhibitors in Ovarian Cancer: The Route to “Ithaca”. Diagnostics (Basel) (2019) 9(2):55. doi: 10.3390/diagnostics9020055 PMC6627688 31109041

[111] Puglisi F Minisini AM De Angelis C Arpino G. Overcoming Treatment Resistance in HER2-Positive Breast Cancer: Potential Strategies. Drugs (2012) 72(9):1175–93. doi: 10.2165/11634000-000000000-00000 22686613

[112] Pernas S Tolaney SM. HER2-Positive Breast Cancer: New Therapeutic Frontiers and Overcoming Resistance. Ther Adv Med Oncol (2019) 11:1758835919833519. doi: 10.1177/1758835919833519 30911337 PMC6425535

[113] Hanker AB Pfefferle AD Balko JM Kuba MG Young CD Sánchez V. Mutant PIK3CA Accelerates HER2-Driven Transgenic Mammary Tumors and Induces Resistance to Combinations of Anti-HER2 Therapies. Proc Natl Acad Sci USA (2013) 110(35):14372–7. doi: 10.1073/pnas.1303204110 PMC3761610 23940356

[114] Nagata Y Lan KH Zhou X Tan M Esteva FJ Sahin AA. PTEN Activation Contributes to Tumor Inhibition by Trastuzumab, and Loss of PTEN Predicts Trastuzumab Resistance in Patients. Cancer Cell (2004) 6(2):117–27. doi: 10.1016/j.ccr.2004.06.022 15324695

[115] Singh JC Jhaveri K Esteva FJ. HER2-Positive Advanced Breast Cancer: Optimizing Patient Outcomes and Opportunities for Drug Development. Br J Cancer (2014) 111(10):1888–98. doi: 10.1038/bjc.2014.388 PMC4229628 25025958

[116] Slamon DJ Leyland-Jones B Shak S Fuchs H Paton V Bajamonde A. Use of Chemotherapy Plus a Monoclonal Antibody Against HER2 for Metastatic Breast Cancer That Overexpresses HER2. N Engl J Med (2001) 344(11):783–92. doi: 10.1056/NEJM200103153441101 11248153

[117] Lui VW Lau CP Ho K Ng MH Cheng SH Tsao SW. Anti-Invasion, Anti-Proliferation and Anoikis-Sensitization Activities of Lapatinib in Nasopharyngeal Carcinoma Cells. Invest New Drugs (2011) 29(6):1241–52. doi: 10.1007/s10637-010-9470-y 20571878

[118] Weroha SJ Oberg AL Ziegler KL Dakhilm SR Rowland KM Hartmann LC. Phase II Trial of Lapatinib and Topotecan (LapTop) in Patients With Platinum-Refractory/Resistant Ovarian and Primary Peritoneal Carcinoma. Gynecol Oncol (2011) 122(1):116–20. doi: 10.1016/j.ygyno.2011.03.030 PMC3104066 21514634

[119] Fortin MA Orlova A Malmström PU Tolmachev V. Labelling Chemistry and Characterization of [90Y/177Lu]-DOTA-ZHER2:342-3 Affibody Molecule, a Candidate Agent for Locoregional Treatment of Urinary Bladder Carcinoma. Int J Mol Med (2007) 19(2):285–91. doi: 10.3892/ijmm.19.2.285 17203203

[120] Pillai MR Chakraborty S Das T Venkatesh M Ramamoorthy N. Production Logistics of 177Lu for Radionuclide Therapy. Appl Radiat Isot (2003) 59(2-3):109–18. doi: 10.1016/S0969-8043(03)00158-1 12941498

[121] Altai M Westerlund K Velletta J Mitran B Honarvar H Karlström AE. Evaluation of Affibody Molecule-Based PNA-Mediated Radionuclide Pretargeting: Development of an Optimized Conjugation Protocol and 177Lu Labeling. Nucl Med Biol (2017) 54:1–9. doi: 10.1016/j.nucmedbio.2017.07.003 28810153

[122] Westerlund K Altai M Mitran B Konijnenberg M Oroujeni M Atterby C. Radionuclide Therapy of HER2-Expressing Human Xenografts Using Affibody-Based Peptide Nucleic Acid-Mediated Pretargeting: *In Vivo* Proof of Principle. J Nucl Med (2018) 59(7):1092–8. doi: 10.2967/jnumed.118.208348 29439013

[123] Tano H Oroujeni M Vorobyeva A Westerlund K Liu Y Xu T. Comparative Evaluation of Novel 177lu-Labeled PNA Probes for Affibody-Mediated PNA-Based Pretargeting. Cancers (Basel) (2021) 13(3):500. doi: 10.3390/cancers13030500 33525578 PMC7865858

[124] Tolmachev V Orlova A. Affibody Molecules as Targeting Vectors for PET Imaging. Cancers (Basel) (2020) 12(3):651. doi: 10.3390/cancers12030651 PMC7139392 32168760

[125] Feldwisch J Tolmachev V. Engineering of Affibody Molecules for Therapy and Diagnostics. Methods Mol Biol (2012) 899:103–26. doi: 10.1007/978-1-61779-921-1_7 22735949

[126] Cong J Liu R Hou J Wang X Jiang H Wang J. Effects of Trastuzumab on the Proliferation and Apoptosis of Ovarian Cancer Cells. Neoplasma (2019) 66(2):240–4. doi: 10.4149/neo_2018_180724N524 30509111

[127] Shang AQ Wu J Bi F Zhang YJ Xu LR Li LL. Relationship Between HER2 and JAK/STAT-SOCS3 Signaling Pathway and Clinicopathological Features and Prognosis of Ovarian Cancer. Cancer Biol Ther (2017) 18(5):314–22. doi: 10.1080/15384047.2017.1310343 PMC5499756 28448787

[128] Hu X Liang Z Zhang C Wang G Cai J Wang P. The Diagnostic Performance of Maximum Uptake Value and Apparent Diffusion Coefficient in Differentiating Benign and Malignant Ovarian or Adnexal Masses: A Meta-Analysis. Front Oncol (2022) 12:840433. doi: 10.3389/fonc.2022.840433 35223521 PMC8864062

[129] Hu X Li D Liang Z Liao Y Yang L Wang R. Indirect Comparison of the Diagnostic Performance of 18F-FDG PET/CT and MRI in Differentiating Benign and Malignant Ovarian or Adnexal Tumors: A Systematic Review and Meta-Analysis. BMC Cancer (2021) 21(1):1080. doi: 10.1186/s12885-021-08815-3 34615498 PMC8495994

[130] Honarvar H Jokilaakso N Andersson K Malmberg J Rosik D Orlova A. Evaluation of Backbone-Cyclized HER2-Binding 2-Helix Affibody Molecule for *In Vivo* Molecular Imaging. Nucl Med Biol (2013) 40(3):378–86. doi: 10.1016/j.nucmedbio.2012.12.009 23357083

[131] Xu T Wang L Jia Y Jia Z Li Z Cui S. Long-Term Multidisciplinary Integrative Therapy Management Resulted in Favorable Outcomes for Ovarian Cancer During Pregnancy: A Case Report and Literature Review. J Ovarian Res (2019) 12(1):108. doi: 10.1186/s13048-019-0584-3 31711529 PMC6844043

[132] Siavoshinia L Jamalan M Zeinali M Pourshohod A Koushki M Moradipoodeh B. Improvement of Targeted Chemotherapy of HER2-Positive Ovarian Malignant Cell Line by ZHER2-Idarubicin Conjugate: An *In Vitro* Study. Iran J Pathol (2021) 16(2):109–18. doi: 10.30699/ijp.2020.120392.2310 PMC8085286 33936221

[133] Altunay B Morgenroth A Beheshti M Vogg A Wong NCL Ting HH. HER2-Directed Antibodies, Affibodies and Nanobodies as Drug-Delivery Vehicles in Breast Cancer With a Specific Focus on Radioimmunotherapy and Radioimmunoimaging. Eur J Nucl Med Mol Imaging (2021) 48(5):1371–89. doi: 10.1007/s00259-020-05094-1 PMC8113197 33179151

