# Supplementary material for: Advances in the Application of Radionuclide-Labeled HER2 Affibody for the Diagnosis and Treatment of Ovarian Cancer
Source: Front Oncol. 2022 Jun 15;12:917439. doi: 10.3389/fonc.2022.917439 (PMC9240272; doi:10.3389/fonc.2022.917439)
Supplement: Supplementary file 1 [file Table_1.doc]

Supplementary Table 1. Detailed amino acid sequence of HER2 extracellular domains Ⅰ-IV.

| HER2 extracellular domain | amino acid sequence |
| --- | --- |
| domain Ⅰ | TQVCTGTDMKLRLPASPETHLDMLRHLYQGCQVVQGNLELTYLPTNASLSFLQDIQEVQGYVLIAHNQVRQVPLQRLRIVRGTQLFEDNYALAVLDNGDPLNNTTPVTGASPGGLRELQLRSLTEILKGGVLIQRNPQLCYQDTILWKDIFHKNNQLALTLIDTN |
| domain Ⅱ | RSRACHPCSPMCKGSRCWGESSEDCQSLTRTVCAGGCARCKGPLPTDCCHEQCAAGCTGPKHSDCLACLHFNHSGICELHCPALVTYNTDTFESMPNPEGRYTFGASCVTACPYNYLSTDVGSCTLVCPLHNQEVTAEDGTQRCE |
| domain Ⅲ | KCSKPCARVCYGLGMEHLREVRAVTSANIQEFAGCKKIFGSLAFLPESFDGDPASNTAPLQPEQLQVFETLEEITGYLYISAWPDSLPDLSVFQNLQVIRGRILHNGAYSLTLQGLGISWLGLRSLRELGSGLALIHHNTHLCFVHTVPWDQLFRNPHQALLHTANRPED |
| domain IV | ECVGEGLACHQLCARGHCWGPGPTQCVNCSQFLRGQECVEECRVLQGLPREYVNARHCLPCHPECQPQNGSVTCFGPEADQCVACAHYKDPPFCVARC |
